# Patient‐specific radiological protection precautions following Cs collagen embedded Cs‐131 implantation in the brain

**DOI:** 10.1002/acm2.13776

**Published:** 2022-09-15

**Authors:** Kavya Prasad, Lawrence T. Dauer, Bae P. Chu, David Aramburu‐Nunez, Gilad Cohen, Kathryn Beal, Brandon S. Imber, Nelson S. Moss

**Affiliations:** ^1^ Department of Medical Physics Memorial Sloan Kettering Cancer Center New York New York USA; ^2^ Department of Radiation Oncology and Brain Metastasis Center Memorial Sloan Kettering Cancer Center New York New York USA; ^3^ Department of Neurological Surgery and Brain Metastasis Center Memorial Sloan Kettering Cancer Center New York New York USA

**Keywords:** brain metastases, brachytherapy, radiation safety

## Abstract

**Objective:**

Cesium‐131 brachytherapy is an adjunct for brain tumor treatment, offering potential clinical and radiation protection advantages over other isotopes including iodine‐125. We present evidence‐based radiation safety recommendations from an initial experience with Cs‐131 brachytherapy in the resection cavities of recurrent, previously irradiated brain metastases.

**Methods:**

Twenty‐two recurrent brain metastases in 18 patients were resected and treated with permanent Cs‐131 brachytherapy implantation using commercially procured seed‐impregnated collagen tiles (GammaTile, GT Medical Technologies). Exposure to intraoperative staff was monitored with NVLAP‐accredited ring dosimeters. For patient release considerations, NCRP guidelines were used to develop an algorithm for modeling lifetime exposure to family and ancillary staff caring for patients based on measured dose rates.

**Results:**

A median of 16 Cs‐131 seeds were implanted (range 6–46) with median cumulative strength of 58.72U (20.64‐150.42). Resulting dose rates were 1.19 mSv/h (0.28–3.3) on contact, 0.08 mSv/h (0.01–0.35) at 30 cm, and 0.01 mSv/h (0.001–0.03) at 100 cm from the patient. Modeled total caregiver exposure was 0.91 mSv (0.16–3.26), and occupational exposure was 0.06 mSv (0.02–0.23) accounting for patient self‐shielding via skull and soft tissue attenuation. Real‐time dose rate measurements were grouped into brackets to provide close contact precautions for caregivers ranging from 1–3 weeks for adults and longer for pregnant women and children, including cases with multiple implantations.

**Conclusions:**

Radiological protection precautions were developed based on patient‐specific emissions and accounted for multiple implantations of Cs‐131, to maintain exposure to staff and the public in accordance with relevant regulatory dose constraints.

## 1 INTRODUCTION

The first surgical implantation of radioactive brachytherapy into a tumor was performed in the early 1900s, parallel to the development of external beam radiotherapy.^1^ These sealed radiotherapy sources were originally developed to deliver high treatment doses to tumors and resection cavities using temporary needle placement, and permanent placements were subsequently popularized. Malignant brain tumor treatment commonly requires upfront or adjuvant irradiation, and brachytherapy has been attempted for these indications since the 1936 description of radon implantation, with subsequent evaluations of iodine‐125(I‐125), which carries a high risk of radiation necrosis due to higher delivered dose (150 Gy), and cesium‐131(Cs‐131), which is posited to have less such risk owing to lower dose deposition of 60 Gy; palladium‐103(Pd103) is commonly used in prostate, ocular melanoma, and other applications but has not been described for brain tumors (Table 1).^2^, ^3^, ^4^, ^5^, ^6^ The use of Cs‐131 has been described for a variety of primary and metastatic brain tumor applications since its FDA approval in 2003. Wernicke et al. implemented a seeds‐on‐a‐strand formulation for resected brain tumors with median seed strength of 2.40U.^7^, ^8^, ^9^, ^10^ More recently, a commercially available formulation of Cs‐131 seeds with 3.50U strength, embedded in a collagen carrier, (GammaTile, GT Medical Technologies, Phoenix, AZ^1^) has been studied.^11^, ^12^, ^13^ The physical half‐life of Cs‐131 is 9.7 days compared to 60 days with I‐125. To deliver the necessary dose (60 Gy to a depth of 5 mm in tissue) and to account for the structural offset built into the tile, a higher seed strength is needed, resulting in higher dose rates over a shorter treatment duration.^14^ Additionally, given larger brain tumor resection cavity sizes and thus higher cumulative seed activity in contemporary use further safety evaluation is warranted.^13^

**TABLE 1:** Comparison of commonly used isotopes in brachytherapy

Isotope	Half‐life (days)	Mean energy (keV)	Exposure rate constant (C‐m^2^/kg‐MBq‐s)	Exposure rate constant (R‐cm^2^/mCi‐h)
Pd‐103	16.99	21	2.73×10^–13^	1.41
I‐125	59.49	28	3.38×10^–13^	1.75
Cs‐131	9.69	30	1.31×10^–13^	0.679

While initial GammaTile reports suggest safety and early efficacy for recurrent meningiomas and a mixed cohort of tumors including in difficult salvage settings, radiation safety considerations with their higher dose rate and seed strength have not been detailed to date.^15^ Prior to widespread adoption of this novel technology, which has been advocated given the importance of early adjuvant radiation for resected brain metastases, it is essential to ensure its safety for patients' surgical and perioperative hospital teams.^16^, ^17^

We describe our initial experience using this new formulation to treat previously irradiated lesions. Specifically, we evaluate radiation safety considerations for patients, intraoperative providers, healthcare workers, and caregivers, and extend this for patients receiving multiple implantations.

## 2 METHODS

### 2.1 Patient inclusion criteria and operative procedures

All prospective patient cases were discussed by a multidisciplinary brain metastasis team of specialists from neurosurgery, neurology, and radiation oncology.^18^ Preoperative magnetic resonance imaging with intravenous gadolinium and perfusion scans were performed in all cases. Resection and placement of brachytherapy was performed by the neurosurgeon under the supervision of the radiation oncologist, who was the authorized user. The tiles were ordered, handled, and prepared by authorized medical physicists who checked the seed strength according to standard hospital procedures to ensure correct dose delivery.

### 2.2 Radiological protection procedures

Patient exposure rates were measured within 48 h postoperatively by the Medical Health Physics team with a Fluke 451B Ion Chamber Survey Meter (Fluke Biomedical, Cleveland, OH), on contact (i.e., patient surface), at 30 cm and at 1 m from the scalp overlying the implantation. The measurements were taken with the ion chamber window open which allowed for reduced attenuation in the setting of low photon energy; correction factors were therefore not needed.^19^ Radiation safety education was provided to patients, family, and staff per institutional procedure via face‐to‐face discussions, written educational materials, door signs, and electronic medical record documentation. Patients were counseled to limit close and prolonged contact with children and pregnant women for a period that depended on the patient's actual exposure readings, as detailed in the remainder of the manuscript.

### 2.3 Modeling exposure to healthcare workers

NVLAP‐accredited (National Voluntary Laboratory Accreditation Program) extremity monitors (ring dosimeters) were provided to all personnel handling the tiles, that is, neurosurgeons, radiation oncologists, and medical physicists preparing the tiles. These readings were corrected for actual background radiation levels using control rings shipped with the exposed rings; when control ring readings were unavailable, the manufacturer subtracted a background‐radiation rate based on our institution's historical average control dose. Estimated radiation exposure to healthcare workers was based on Release Equation (1) where the time of patient interaction was conservatively estimated to be 6 h within 1 m over a 3‐day postoperative hospitalization, assuming an exposure rate constant 1.31×10^−13^ C m^2^/kg MBq s or 0.679 R cm^2^/mCi‐h.^14^, ^20^

### 2.4 Modeling exposure to members of the public and precaution time

Family member/visitor exposures were calculated using a modified version of Release Equations (1) and (2) from NRC Regulatory Guide 8.39.^21^

(1) $$\begin{array}{ccc} E_{t} & = & 34.6\;\times 0.00876\times T_{r}\times T_{p}\times X_{r,0}\times {\left(\frac{E}{\left(K_{a}\right)}\right)}_{r}\\ & & \times \left(1-e^{-\frac{\ln2\times t}{T_{p}}}\right) \end{array}$$

(2) $$E_{t}=34.6\;\times 0.00876\times T_{r}\times T_{p}\times X_{r,0}\times {\left(\frac{E}{\left(K_{a}\right)}\right)}_{r}$$

where, *X*_r,0_ = exposure rate at distance *r* in mR/h, *T_r_* = occupancy factor for different activities, *T_p_* = physical half‐life in days, and t = time precautions taken in days.

Equation (1) estimates exposure as a function of time and Equation (2) calculates lifetime exposure, described by the United States Nuclear Regulatory Commission below, modified to use real‐time patient‐specific exposure rates and occupancy factors specific to varying day‐to‐day situations. Patient precaution times, defined as period of complete exposure avoidance, were modeled on the algorithm provided by Dauer et al. for prostate seed implants using dose rate measurements at 1 ft.^22^ Values for energy and yield‐weighted (*E*/*K_a_*)*_r_* (effective dose per unit air kerma conversion factor for index distance *r* based on the exposure situation [mSv/mGy]) conversion factors were calculated based on the American Association of Physicists in Medicine and International Commission on Radiological Protection‐recommended data for Cs‐131.^23^ Anteroposterior irradiation geometry (AP) *E*/*K_a_* values were used for five different situations: non‐pregnant sleeping adult, pregnant sleeping adult, child held occasionally where rotational irradiation geometry (ROT) *E*/*K_a_* values were used, non‐pregnant visitors, pregnant/child visitors, and the public. Based on Equation (2), a plot of exposure rate versus precaution duration with logarithmic best‐fit equations was generated. As Low as Reasonably Achievable (ALARA) guidelines of 50% of the annual limit of 1 and 5 mSv were chosen conservatively to facilitate an algorithm that allows for multiple implants.^21^ Different ALARA guidelines can be chosen to allow for varying number of treatments.

A “shielding factor,” or ratio of the calculated dose rate from an unshielded Cs‐131 source at 1 m to the measured dose rate from the patient at 1 m, was calculated to inform the attenuation due to the intervening cranium, brain, and other soft tissue.

$$\begin{array}{ccc} & & \mathrm{Shielding}\;\mathrm{Factor}\;\mathrm{SF}\\ & & \;=\frac{\mathrm{Dose}\;\mathrm{rate}\;\mathrm{from}\;\mathrm{unshielded}\;\mathrm{point}\;\mathrm{source}\;\mathrm{at}\;1m}{\mathrm{Measured}\;\mathrm{dose}\;\mathrm{rate}\;\mathrm{from}\;\mathrm{patient}\;\mathrm{at}\;1m\;} \end{array}$$

Illustrated below is an example shielding factor calculation for patient 1 who received a total implanted activity of 3180 MBq, resulting in calculated unshielded point dose rate of 0.0584 mSv/h and measured patient dose rate of 0.0062 mSv/h leading to a shielding factor of 9.4.

$$\mathrm{SF}=\frac{5.84}{0.62}=9.4$$

## 3 RESULTS

Eighteen patients underwent twenty‐two implantations, including two patients who received multiple treatments (Table 2).

**TABLE 2:** Patient and operating room staff exposures

Case ID	Number of seeds	Total seed strength (U)	Total activity (MBq)	Dose rate (mSv/h)			Ring dose (mSv; corrected)	
				Contact	30 cm	1 m	Operative team (highest)	Control
1^a^	16	55.52	3180	0.71	0.054	0.0062	0.06	0.18
2	16	56.00	3208	0.33*	0.027*	0.0067*	n/a	n/a
3^a^	16	55.84	3198	0.28	0.056	0.0098	n/a	n/a
4	20	69.40	3974	1.30	0.097	0.0150	0.09	0.08
5	16	60.96	3492	1.46	0.133	0.0127	0.02	0.10
6	24	84.00	4811	0.54	0.054	0.0193	0.08	0.10
7	28	98.00	5613	1.52	0.139	0.0230	0.08	0.10
8^b^	12	42.36	2426	1.32	0.104	0.0163	0.04	0.05
9	20	71.40	4090	1.01	0.129	0.0169	0.19	0.21
10	18	61.87	3543	0.79	0.086	0.0086	n/a	n/a
11^a^	22	76.78	4398	1.70	0.098	0.0194	0.52	0.12
12	6	20.64	1182	0.40	0.039	0.0040	n/a	n/a
13	14	48.16	2758	1.42	0.129	0.0145	0.57	0.24
14^a^	24	83.28	4770	1.22	0.129	0.0120	0.57**	0.06
15	16	56.32	3226	1.17	0.053	0.0113		0.06
16	10	35.30	2022	1.29	0.139	0.0104	0.12	0.1***
17	16	56.48	3235	1.52	0.059	0.0089	0.27	
18	20	70.80	4055	1.55	0.062	0.0081	0.09	
19	46	150.42	8615	3.30	0.350	0.0390	0.45	0.2
20	22	76.12	4360	1.46	0.075	0.0081	0.29	0.12***
21	16	55.68	3189	0.58	0.060	0.0064	0.12	
22^b^	12	42.36	2426	1.14	0.091	0.0102	0.28	

* These measurements were taken on postoperative day 2 and corrected for decay.

** This represents the combined measurement from cases 14 plus 15 as the same ring was worn for both cases, disallowing calculation of a median value.

*** Same control used for these cases.

^a^ Same patient.

^b^ Same patient.

Abbreviations: MBq, megabecquerel; mSv, millisievert.

Median air kerma strength was 3.50U/seed with a variance of 0.09U, except for one patient (Case 5) who underwent surgery with brachytherapy placement 1 day sooner than originally planned, resulting in a per‐seed strength of 3.81U and an expected dose of 65.3 Gy at 5 mm from the cavity surface. The median number of implanted Cs‐131 seeds was 16(range: 6–46) with median total implant strength of 58.72U^2^ (range: 20.64–150.42).

### 3.1 Radiation safety considerations

**FIGURE 1:**
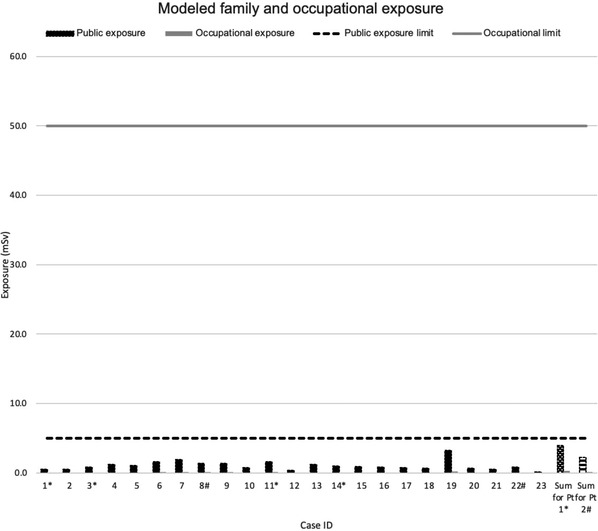
Modeled exposure for healthcare workers and the family members or caregivers regulatory limits reflect exposure limits of 50 mSv/year for medical personnel and 5 mSv/year for members of the caregivers. Cases denoted with (*) reflect the same patient who underwent four implantations, and (#) a patient who underwent two implantations.

The median effective dose rates were 1.25 mSv/h (0.28–3.30) on contact, 0.09 mSv/h (0.03–0.35) at 30 cm and 0.01 mSv/h (0.0017–0.039) at 1 m from the patient. The corrected median ring dose was 0.04 mSv (0–0.19) for the radiation oncologist, 0.09 mSv (0–0.57) for the neurosurgeon, and 0.27 mSv (0–0.52) for the neurosurgical fellow/resident (Table 2). Modeled lifetime family‐member exposure was 0.91 mSv (0.33–3.26), and healthcare worker exposure was 0.06 mSv (0.02–0.23), all well below regulatory limits (Figure 1 and Table 3).

**TABLE 3:** Modeled healthcare worker and family exposures

Patient ID	Total activity (MBq)	Calculated unshielded dose rate at 1 m (mSv/h)	Shielding factor	Calculated lifetime family exposure (mSv)	Calculated 3‐day personnel exposure (mSv)
1*	3180	0.06	9.4	0.5	0.04
2	3208	0.06	8.8	0.6	0.04
3*	3198	0.06	6.0	0.8	0.06
4	3974	0.07	4.9	1.3	0.09
5	3492	0.06	5.0	1.1	0.08
6	4811	0.09	4.6	1.6	0.11
7	5613	0.10	4.5	1.9	0.14
8 ^#^	2426	0.04	2.7	1.4	0.10
9	4090	0.08	4.4	1.4	0.10
10	3543	0.07	7.6	0.7	0.05
11*	4398	0.08	4.2	1.6	0.10
12	1182	0.02	5.4	0.3	0.02
13	2758	0.05	3.5	1.2	0.09
14 *	4770	0.09	7.3	1.0	0.07
15	3226	0.06	5.2	0.9	0.07
16	2022	0.04	3.6	0.9	0.06
17	3235	0.06	6.7	0.7	0.05
18	4055	0.07	9.2	0.7	0.05
19	8615	0.15	4.1	3.3	0.23
20	4360	0.08	9.9	0.7	0.05
21	3189	0.06	9.1	0.5	0.04
22^#^	2426	0.04	4.4	0.9	0.06

* Same patient.

^#^ Same patient.

### 3.2 Patients with multiple craniotomies or extreme exposure

We highlight extreme exposure examples for the purposes of confirming this strategy's safety for patients and providers. There were two patients with multiple craniotomies. One patient was treated with four sequential craniotomies (cases 1, 3, 11, and 14). By the time of implantation #4, the first two implantations were assumed to be fully decayed (>10 half‐lives). Implant #3 was not considered to be fully decayed (<5 half‐lives) and intraoperative radiation exposure measurements were taken to evaluate precautions following the fourth resection. The integrated dose from all four implantations was 3.97 mSv to family members, below the annual regulatory limit of 5 mSv. In another patient with two sequential craniotomies (cases 8 and 22), the first implantation was taken to be fully decayed by the time of the second resection, keeping the integrated dose to 2.2 mSv.

Additional evaluations were also performed for patient #19 who received brachytherapy placements in a single surgery to two tumor cavities with a total of 46 seeds, and a cumulative seed strength of 150.4 U or 8620 MBq (232.9 mCi). Although exposure rates of 3.3 mSv/h on contact and 0.039 mSv/h at 1 m resulted in overall exposure below regulatory limits, the patient was conservatively prescribed 3 weeks of precautions for adult family members and visitors, and 6 weeks of precautions for pregnant visitors and children.

In the 22 cases reconstructed with native cranium, the mean shielding factor was significantly higher for infratentorial than supratentorial cases (8.28 ± 1.0 vs 4.8 ± 0.7, p < 1 × 10^5^; Table 3). In the one case reconstructed with mesh titanium (#8), the lowest shielding factor of the cohort was identified (2.7); this did not change duration of precautions substantially.

### 3.3 Algorithm for calculating duration of precautions

*E*/*K_a_* qnteroposterior and rotational geometry were calculated using the photon energy abundance based on ICRU Report 57 recommendations resulting in *E*/*K_a_* AP 0.366 mSv/mGy and ROT 0.166 mSv/mGy.^23^ An abundance weighted mean photon energy for Cs‐131 was calculated followed by looking up corresponding AP and ROT values as described in Table 4.

**TABLE 4:** Energy spectrum of Cs‐131

Energy	Abundance	Weighted energy
29.45	0.213	7.81
29.78	0.395	14.64
33.57	0.037	1.55
33.63	0.0718	3.00
34.4	0.00777	0.33
34.41	0.0121	0.52
34.54	0.000796	0.03
34.55	0.00151	0.06
4.413	0.0217	0.12
4.097	0.0374	0.19
4.707	0.00547	0.03
	0.803546	28.29

Example: For a 29.45 keV photon with abundance of 0.213 the weighted energy was 29.45 × 0.213 = 7.81 keV. Once performed for all photon energies, this was summed up to yield effective energy of 28.29 keV. AP and ROT values for 28.29 keV are 0.366 and 0.166 mSv/mGy.

**FIGURE 2:**
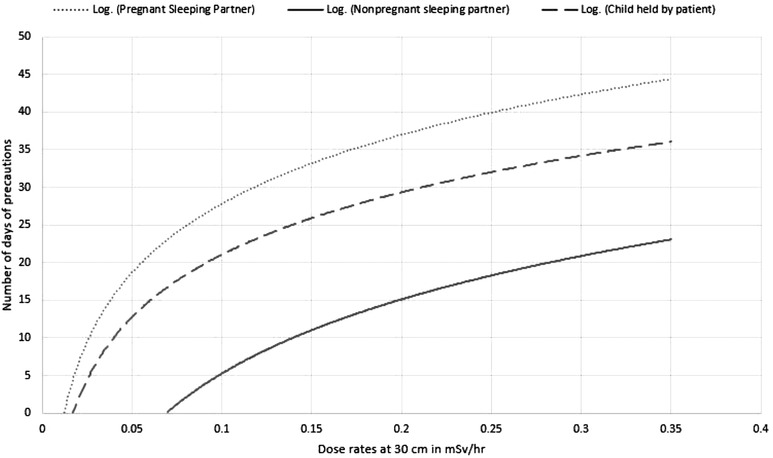
Dose rate‐based precaution guidelines. Exposures at 30 cm are described for three specific situations: non‐pregnant sleeping partner (solid line), pregnant sleeping partner (dotted line), and child held occasionally with the assumption that these situations typically involve separation of 30 cm and anterior–posterior geometry (dashed line). These logarithmic best‐fit curves represent precaution time as a function of measured dose rates.

Effective lifetime doses exceeding the chosen ALARA guidelines after *t* days of precautions were calculated using the formulation described above and similar data was calculated for varying dose rates from 0.01 to 0.35 mSv/h at 30 cm for sleeping partners and children held in the lap, and 0.0025 to 0.04 mSv/h at 1 m for members of the public and other visitors. For one cycle of treatment, the full annual limit of 1 and 5 mSv were assumed, for two cycles 50% of that, 0.5 and 2.5 mSv, for four cycles 25% and so on. For each of the dose rates and adjusted exposure limits, the minimum duration of precautions was calculated, which was plotted against dose rates. The equations in Table 5 are a result of the logarithmic fit to the plots. An example of the plot for two cycles of treatment is illustrated in Figure 2.

Dose rates at 1 m were used for the remaining situations: non‐pregnant visitors, pregnant and child visitors, and members of the public with the assumption that these situations typically involve separation of 1 m and ROT geometry. For these exposure situations the dose to total decay was less than 50% of the chosen ALARA guideline at time *t* = 0; thus, based on our cohort, these situations do not need any precautions even at the highest measured dose rates. While we estimate it is unlikely that an implant will exceed regulatory exposure limits, our experience with such large implants is limited to one case. We therefore recommend that precautions for patient with large implants be done on a case‐by‐case basis.

Illustrated in Table 5 below, we have generated the equations based on this algorithm for one, two, four, and five cycles of resection with a total annual exposure limit of 5 mSv for non‐pregnant sleeping partners and 1 mSv for pregnant sleeping partners and children. The same method can be applied to *n* number of cycles to achieve a specific target annual exposure limit.

**TABLE 5:** Generated algorithm to calculate duration of precautions (days) for *N* resections with GammaTile placements in one year based on measured postoperative dose rate, *X* (mR/h), at 30 cm

Exposure scenario	*N* = 1	*N* = 2	*N* = 4	*N* = 5
Non‐pregnant sleeping partner	14.119 ln(*x*) − 37.065	14.255 ln(*x*) − 27.551	13.873 ln(*x*) − 16.687	14.252 ln(*x*) − 14.641
Pregnant sleeping partner	11.505 ln(*x*) − 7.7138	12.913 ln(*x*) − 2.1746	10.435 ln(*x*) + 8.1494	13.942 ln(*x*) + 8.247
Child held occasionally	12.414 ln(*x*) − 19.914	11.731 ln(*x*) − 6.0811	10.433 ln(*x*) + 1.0577	13.957 ln(*x*) + 1.2627

## 4 DISCUSSION

We describe our initial institutional application of recently introduced commercially available GammaTile Cs‐131 implants for brain tumors.

In our study, extremity dose to staff handling the tiles was sufficiently low to abrogate the need for lead gloves/aprons during implantation.^24^ The highest intraoperative extremity exposure was 0.57 mSv, roughly 0.1% of the annual limit.^21^ The resident/fellow typically received higher extremity doses, attributed to the additional proximity and duration of exposure during closing. Additionally, extensive training was provided to staff caring for the patients postoperatively to ensure their exposure remained below recommended limits. Our modeled staff exposure suggested this paradigm was successful.

Shielding factors demonstrated additional shielding offered by infratentorial implantation as opposed to supratentorial placements which we ascribe to the additional buffer of overlying nuchal muscle coverage. Shallower placements resulted in much higher external exposure rates compared to deeper implants.

We also present evidence‐based recommendations for customized precautions for various situations based on measured dose rates and suggest a maximum of 50% of the annual dose limit guidance for patient release, affording flexibility for future implantation while maintaining safety and regulatory compliance. This is important in patients with oligometastatic CNS disease which is a common scenario.^6^ Notably, in the case of a patient who received four implantations, total caregiver exposure was estimated to be 26% less than the annual limit, despite the large sizes of these metastases.^21^ This dose rate‐based algorithm eliminates the variability and uncertainty associated with educating families on necessary precautions. While this work describes the development of a radiation protection protocol designed specifically for Cs‐131 brain implants, this methodology can be extended to other applications such as the implantation of metastatic lesions and lymph nodes using I‐125 or Pd‐103.

Limitations of this study include the relatively small patient cohort. Additional larger‐scale prospective studies with additional follow‐up are necessary to establish more definitive safety and efficacy data and is the basis for an ongoing phase 2 randomized trial (NCT04690348).

## 5 CONCLUSIONS

In early experience with detailed radiation safety evaluation, salvage intracavitary Cs‐131 implantation employed for large recurrent brain metastases was associated with a favorable radiation safety profile for patients, operating room staff and other hospital providers, and caregivers.

## AUTHOR CONTRIBUTIONS

Kavya Prasad: Primary author, contributions to the conception and design, data acquisition, data analysis, development of algorithm.

Lawrence T. Dauer: final approval of the work, accuracy and integrity

Bae P. Chu: final approval of the work, accuracy and integrity

David Aramburu‐Nunez: contributions to the conception and design, implementation of brachytherapy program, final approval of the work, accuracy and integrity

Gilad Cohen: contributions to the conception and design, implementation of brachytherapy program, final approval of the work, accuracy and integrity, interpretation of data

Kathryn Beal: contributions to the conception and design, implementation of radiation oncology program in theory and in operating room, final approval of the work, accuracy and integrity

Brandon S. Imber: implementation of radiation oncology program in theory and in operating room, final approval of the work, accuracy and integrity

Nelson S. Moss: Primary author, contributions to the conception and design, implementation of trial in operating room, final approval of the work, accuracy and integrity

## ETHICS STATEMENT

The authors state that they have obtained appropriate institutional review board approval or have followed the principles outlined in the Declaration of Helsinki for all human investigations. In addition, informed consent has been obtained from the participants involved. This work is compliant with CARE guideline.

## Data Availability

Not applicable.

## Footnotes

1 GammaTile GT Medical Technologies https://gtmedtech.com/

2 U or air kerma strength as defined in TG‐43U1 is the air‐kerma rate *K*˙*δ*(*d*), in vacuo, due to photons of energy greater than *δ* at a distance *d*, multiplied by the square of this distance, *d* ^2^.
